# Application of Time-Scale Decomposition of Entropy for Eye Movement Analysis

**DOI:** 10.3390/e22020168

**Published:** 2020-02-01

**Authors:** Katarzyna Harezlak, Pawel Kasprowski

**Affiliations:** Silesian University of Technology, Akademicka 16, 44-100 Gliwice, Poland; pawel.kasprowski@polsl.pl

**Keywords:** eye movement events detection, nonlinear analysis time series analysis, approximate entropy, fuzzy entropy, multilevel entropy map, time-scale decomposition

## Abstract

The methods for nonlinear time series analysis were used in the presented research to reveal eye movement signal characteristics. Three measures were used: approximate entropy, fuzzy entropy, and the Largest Lyapunov Exponent, for which the multilevel maps (MMs), being their time-scale decomposition, were defined. To check whether the estimated characteristics might be useful in eye movement events detection, these structures were applied in the classification process conducted with the usage of the kNN method. The elements of three MMs were used to define feature vectors for this process. They consisted of differently combined MM segments, belonging either to one or several selected levels, as well as included values either of one or all the analysed measures. Such a classification produced an improvement in the accuracy for saccadic latency and saccade, when compared with the previously conducted studies using eye movement dynamics.

## 1. Introduction

Biological signals representing the electrical, chemical, and mechanical activities that occur during biological events attracted the attention of many researchers. These interests are aimed at discovering patterns which may prove useful in understanding the underlying physiological mechanisms or systems. The range of biological signals explored in various studies include, inter alia: electroencephalogram (EEG), electrocardiogram (ECG), surface electromyogram (sEMG), galvanic skin response (GSR), and arterial blood pressure (ABP). Obtaining these characteristics is of great importance in medicine, as this it may enable the differentiation of typical and atypical behaviors, supporting in this way diagnosing and treatment. However, analysis of some of them may also be applied, for example, in cognitive or psychological studies.

Acquiring bio-signal recordings requires various biomedical instruments to be applied. For example, eye movement signals, which are of interest in this article, can be measured by means of the electrical potential between electrodes placed at points close to the eye (electro-oculography (EOG)) or with the usage of less intrusive video-oculography (VOD), using video cameras and image processing algorithms for eye movement tracking.

Biological signals are naturally analogous; however, during measurement process conducted with a specific sampling rate, they are converted to a discrete-time form and constitute a biological time series. The distinct difficulty in the analysis of bio-signals is their nonlinear nature, therefore in order to extract useful features and components of the recorded signal, it is important to use appropriate processing methods. Frequently, methods of nonlinear time series analysis, which can be deployed for many types of bio-signals, are chosen for this purpose. Some of these are described in the following section.

### 1.1. Methods Used in Biological Signal Analysis

The approaches used for quantifying biological signal characteristics use fractal and dynamic feature analysis. In the former group of traits, fractal power spectra and the fractal dimension are investigated, usually, through detrended fluctuation analysis (DFA) [1]. This approach enables the assessment of the existence of self-similarity and long-range correlations. The main goal of these kinds of studies is revealing the long-term memory of an explored system and predicting the system’s future states. This method was successfully applied to such biological processes as ECG, EEG, EMG [2,3,4,5].

Nonlinear system dynamics may also be represented by various entropy measures [6], and the Largest Lyapunov Exponent (LLE) [7]. The entropy-based algorithms ascertain the degree of disorder and uncertainty in the underlying system described by an observed variable. Two primary purposes of these studies can be mentioned. The first of them is the discovery of the dynamic characteristics of biological signals of healthy people to use them as reference data in revealing anomalous cases. The second one is to determine events occurring within these signals.

For example, Chen et al. in [8] provided a review of entropy measures used in cardiovascular diseases. Furthermore, *approximate entropy* (ApEn) was used in [9] to obtain a better understanding of abnormal dynamics in the brain in the case of Alzheimer’s disease (AD). Yents et al. [10] used spatio-temporal gait data from young and elderly subjects to investigate the performance of ApEn and *sample entropy* (SampEn). Cao et al. [11] presented the *fuzzy entropy* (FuzEn) metrics comparison and pointed the FuzEn out as a better tool than ApEn and SampEn, for distinguishing EEG signals of people with AD from those obtained for non-AD. Additionally, the comparison of various fuzzy entropy measures was conducted in the work [12]. Some synthetic datasets, together with EEG and ECG clinical datasets, as well as the gait maturation database, were used for comparison purposes.

EEG, ECG, and gait characteristics were also explored with the usage of the LLE. The works conducted in [13,14] indicated that this measure can be used to identify sleep stages, whereas in [15] the LLE was applied for the analysis of nonlinear changes of neural dynamics in the processing of stressful anxiety-related memories. Furthermore, automated diagnosis of electrocardiographic changes was successfully explored in [16] by using the Lyapunov Exponents for the detection of four types of ECG beats. Additionally, some works were devoted to investigating the application of the LLE in human locomotion for gait stability assessment. For example, Josinski et al., in their research [17] focused on finding LLE-based, easy-to-measure, objective biomarkers that could classify PD (Parkinson’s disease) patients in early (preclinical) stages of the disease.

Nonlinear analysis was also conducted in regard to eye movement signal. However, its dynamics in this scope was not so widely explored as in other biological signals. Nonetheless, in several studies, it was discovered, based on the LLE [18,19,20] and entropy measures: ApEn and SampEn ([21,22]), that various phases of eye movement signal reveal different dynamic characteristics.

### 1.2. Dynamic Eye Movement Signal Characteristics

**Figure 1:**
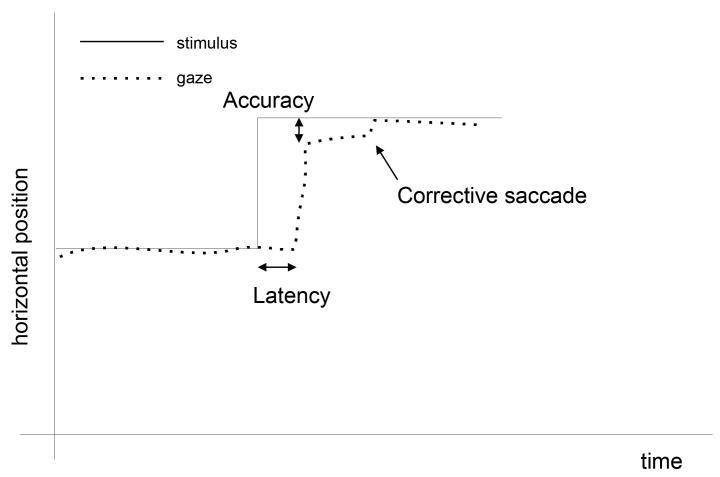
An example of eye movement events. At the beginning the eye is focused on the stimulus. When the stimulus moves to another location, the eye moves as well, yet after a period of saccadic latency [22].

Eye movements are triggered by brain activity, which is a response to visual stimuli or an intention to gain knowledge of the surrounding world. The biological signal evoked this way consists of two main components: fixations, occurring when eyes are focused on part of a scene, and saccades taking place when eyes move to another scene area. During both events, eyes are in constant motion, even during fixations, generating micro-movements such as tremors, microsaccades, and drifts [23,24]. Additionally, in the reaction to the observed stimulus movement, the brain needs some time to commit a saccade. This occurrence called *saccadic latency* [25] is shown, together with the preceding fixation and the following saccade, in Figure 1.

Fixation and saccade differ in their characteristics. The first element is described by slow motions, small spatial dispersion, and relatively long duration (200–300 ms up to several sec.), while during the second one eyes move very quickly, reaching up to even $500^{\circ }$/s, with a short duration (30–80 ms) and a higher amplitude of movement [26]. These differences are of prominent importance when event detection algorithms are considered. The most common approaches for extracting fixations and saccades from registered signal use dispersion and velocity thresholds [27]. One of the major drawbacks of these solutions is the dependency of the obtained results on user-defined thresholds and the lack of their commonly accepted values. The alternative approach presented in [28] assumes the use of machine learning and 14-features vector constructed based on the 100–200-ms surroundings of each sample. This vector consists of features describing the data in terms of dispersion, velocity, sampling frequency, and precision. The results of these studies showed that the machine learning technique is a promising solution. It was taken into consideration when a new approach was being developed using the findings of the previously described nonlinear dynamic systems analysis. For eye movement events detection, in [22], a multilevel map of ApEn was defined, and entropy values calculated at each map level were used for the feature vectors construction. Determining particular eye movement segments was conducted using the kNN classifier for various values of the k parameter. The classification outcomes were promising as well, especially in the scope of saccadic period detection, confirming the usefulness of ApEn measure in such an application. However, because one measure is only a single index describing the behavior of a dynamic time series, further investigations are still required. The currently presented research is the answer to this need.

### 1.3. Contribution

These studies introduce an extended application of the multilevel map by adding two additional measures: FuzEn and the LLE. According to the authors’ knowledge, the fuzzy entropy has not been applied in exploring eye movement dynamics so far. The idea of the application of the multilevel map, built based on FuzEn and the LLE, for eye movement time series analysis is also a novel approach, as well as combining three multilevel maps in order to define feature vectors for machine learning eye movement event detection.

## 2. Materials and Methods

### 2.1. The Dataset Description

The presented research was conducted with the usage of the same dataset, which was applied in the studies described in [22]. It was collected during the”jumping point” experiment, using 29 dark points ($N_{pp}$), distributed over a white 1280×1024 (370 mm × 295 mm) screen. The points’ layout was designed in such a way to ensure both covering a screen area evenly and to obtain varying lengths of saccadic movements. Although the point was displayed in each location for 3 sec, only the first $N_{EMr}$ = 1024 ms were taken into account during further analysis. Eye movement signals were acquired employing the head-mounted JAZZ-novo eye tracker [29], with a sampling rate equal to 1000 Hz. It uses Direct Infra Red Oculography (IROG), which is embedded in the Cyclop ODS sensor measuring the resultant rotations of the left and the right eye. During registration, eyes are illuminated with a low intensity infrared (IR) light. The difference between the amounts of IR reflected back from the eye surfaces, carries information pertaining to the eye position changes.

24 participants ($N_{p}$) aged between 22 and 24, with normal vision, took part in the experiment, which consisted of two sessions, separated by three weeks ($N_{spp}$). As a result,$N_{EMs}$ participants’ sessions were gathered, $N_{ps}=N_{p}*N_{spp}$, each of which comprised subsequent eye positions for all points’ locations. Such a dataset was divided into $N_{EMs}$ eye movement series ($N_{EMs}=N_{p}*N_{spp}*N_{pp}$), which are later referred to as *EM series*. Each of them included one participant’s eye positions registered between the moment of the appearance of the stimulus and the time of its position change. Due to some registration and processing problems four participant session were removed from further analysis. Thus, the $N_{ps}$ value decreased by 4, and $N_{EMs}$ by $4\times N_{pp}$. Finally, $N_{EMs}$—the number of eye movement time series—was 1276 (44participants_sessions×29points)

Subsequently, by applying the standard procedure of the two-point signal differentiation, the first derivative of horizontal coordinates for each EM series was obtained. Thus, each series corresponds to the velocity of eye movement in a period between two appearances of the stimulus. This quantity was used to make research independent to the point positions.

### 2.2. The Method

**Figure 2:**
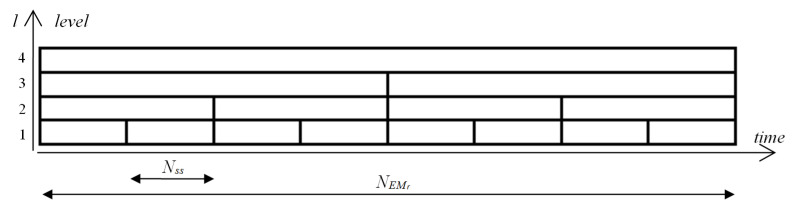
Segments in the multilevel time-scale structure.

The first step of the nonlinear time series analysis was the evaluation of the three chosen measures: ApEn, FuzEn and the LLE, for each EM series. These calculations were conducted taking into account the structure of the multilevel map (MM) introduced in [22]. It represents the concept of a time-scale decomposition of the particular measure, whose values are calculated for various segments of EM series following the schema presented in Figure 2.

A signal at the (l+1)-th level of the map is divided into two shorter segments at the *l*-th level. The smallest size of segments was used at the 1st level of the map, and for this research, it was set to $N_{ss}$=64 ms. As mentioned, the time series length $N_{EMr}$ was set to 1024 ms, which resulted in l=5 map levels. This means that at the 2nd level the length of a segment equaled 128 ms, at the 3rd: 256 ms, at the 4th: 512 ms and finally at the 5th: 1024 ms. For each map cell, all three measures were estimated independently; thus, three MMs were obtained for each EM series (Figure 4).

### 2.3. Approximate Entropy

The approximate entropy method was proposed in [30] as a measure of regularity for quantifying complexity within a time series. For a given integer parameter *m* and a positive real one *r*, for a time series u(1),…,u(N) in **R**, the sequence of vectors x(1),…,x(N−m+1) in **R**${}^{m}$ can be defined, where x(i)=[u(i),…,u(i+m−1)].

For 1≤i≤N−m+1, using probability that any two vectors of length *m* are similar within a tolerance given by *r*:

(1) $$C_{i}^{m}\left(r\right)=\frac{\mathrm{number}\;\mathrm{of}\;j\leq N-m+1:d[x(i),x(j)]\leq r}{N-m+1}$$

where the distance *d* is defined as follows:

(2) $$d\left[x\left(i\right),x\left(j\right)\right]=\max_{k\in (1,m)}(|u\left(i+k-1\right)-u\left(j+k-1\right)|)$$

and the average logarithmic probability over all m-patterns:

(3) $$\Phi^{m}\left(r\right)={\left(N-m+1\right)}^{-1}\sum_{i=1}^{N-m+1}\log C_{i}^{m}\left(r\right)$$

approximate entropy can be obtained:

(4) $$\mathrm{ApEn}\left(m,r\right)=\lim_{N\to \infty }\left[\Phi^{m}\left(r\right)-\Phi^{m+1}\left(r\right)\right].$$

The asymptotic formula in Equation (4) cannot be used directly, and the estimator of approximate entropy (for sufficiently large values of *N*) is defined as:

(5) $$\mathrm{ApEn}\left(m,r,N\right)=\Phi^{m}\left(r\right)-\Phi^{m+1}\left(r\right).$$

There is usually significant variation in ApEn(m,r,N) over the range of *m* and *r*, when a particular system is taken into consideration [31]. Thus, based on the analysis conducted in [22], in this research *m* was set to 2 and *r* as 0.2×SD (the standard deviation of the entire time series) in order to define the first MM.

### 2.4. Fuzzy Entropy

The second MM was prepared based on the fuzzy entropy values calculated according to the method introduced in [32], which consists of several steps.

For a given time series u(1),…,u(N) and integer parameter *m*, the sequence of vectors $X_{1}^{m},\ldots ,x_{\left(N-m+1\right)}^{m}$ is defined as:

(6) $$X_{i}^{m}=u(i),\ldots ,u(i+m-1)-u0\left(i\right)$$

where $X_{i}^{m}$ represents *m* consecutive *u* values, commencing with the i−th point and generalized by removing a baseline:

(7) $$u0\left(i\right)=\frac{1}{m}\sum_{j=0}^{m-1}u\left(i+j\right)$$

Subsequently, the distance between two vectors is defined as:

(8) $$d_{ij}^{m}=d\left[X_{i}^{m},X_{j}^{m}\right]=\max_{k\in (0,m-1)}\left|(u(i+k)-u0(i))-(u(j+k)-u0(j))\right|$$

and given *n* and *r* the similarity degree $D_{ij}^{m}$ of $X_{j}^{m}$ to $X_{i}^{m}$ through a fuzzy function $\mu (d_{ij}^{m},n,r)$ is calculated:

(9) $$D_{ij}^{m}=\mu \left(d_{ij}^{m},n,r\right)$$

where:

(10) $$\mu \left(d_{ij}^{m},n,r\right)=exp\left(-\frac{{\left(d_{ij}^{m}\right)}^{n}}{r}\right)$$

Finally, the fuzzy entropy for finite sets is estimated as:

(11) $$FuzEn\left(m,n,r,N\right)=\ln\phi^{m}\left(n,r\right)-\ln\phi^{m+1}\left(n,r\right)$$

where

(12) $$\phi^{m}=\frac{1}{N-m}\sum_{i=1}^{N-m}\left(\frac{1}{N-m-1}\sum_{j=1,j\neq i}^{N-m}D_{ij}^{m}\right)$$

*N* corresponds to the size of the time series, which depends on the MM level under consideration. *m* is the length of sequences to be compared and was set to the same value as for evaluating approximate entropy. The parameters *n* and *r*, determining the width and the gradient of the boundary of the exponential function, respectively, were chosen based on experimental data, following suggestions provided by the method’s authors. Fuzzy entropy was evaluated for 20 time series with different *r* and *n* values. Subsequently, standard deviation (SD) of the results was calculated. The parameter values for which a slow decrease in SD was observed were chosen for the purpose of these studies. They were 2 for *n* and 0.075×SD (of the particular EM series) for *r*.

### 2.5. The Largest Lyapunov Exponent

The Largest Lyapunov Exponent is a measure which estimates the amount of chaos in dynamic systems. Chaos is observed when neighbouring paths followed by the system, starting from very close initial conditions, rapidly—exponentially fast—move to different states. The reconstruction of the system states is feasible based on measurements of a single observed property when Takens’ embedding theorem is applied [33]. A time delay embedded series—with time lag denoted by τ and embedding dimension by *m*—can be defined as a transformation of the original time series *u*, such that:

(13) $$x\left(i\right)=\left[u\left(i\right),u\left(i+\tau \right),\cdots ,u\left(i+\left(m-1\right)\tau \right)\right],i=\left\{1,2\cdots ,M\right\}$$

where M=N−(m−1)×τ.

A pair [x(i),x(j)] of nearest neighbours, starting close to one another in a chaotic system, diverges approximately at a rate given by the Largest Lyapunov Exponent λ [34]:

(14) $$d_{j}\left(i\right)\approx d_{j0}e^{\lambda (i\Delta t)}$$

where $d_{j}\left(i\right)$ is the Euclidean distance after *i* time steps, Δt is the sampling rate of the time series and $d_{j0}$ is the initial pair separation. Solving the Equation (14) by taking the logarithm of both sides, the Largest Lyapunov Exponent can be calculated as follows:

(15) $$\lambda \approx \frac{1}{i\Delta t}ln\left(\frac{d_{j}\left(i\right)}{d_{j0}}\right)$$

Equation (15) provides the way for evaluating λ for two specific neighbouring points over a specific interval of time. Thus, to approximate the Lyapunov Exponent for a whole dynamic system, it has to be averaged for all j=1,2,…,M, where M=N−(m−1)×τ. Negative Lyapunov Exponent values indicate convergence, while positive demonstrate divergence and chaos. The parameters *m* and τ are calculated with the usage of the False Nearest Neighbours (FNN) [35] and Mutual Information Factor [36], respectively. The above-described methods were used in the current research to define the third MM.

### 2.6. Detection of Eye Movement Events

**Figure 3:**
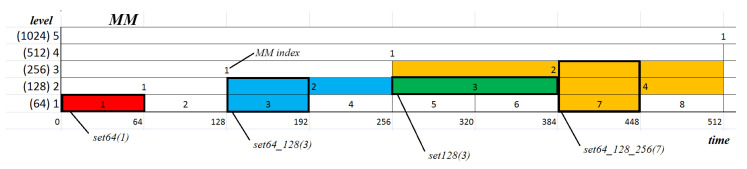
Sample one-, two-, and three-dimensional feature vectors embedded in the MM structure [22].

Following the studies presented in [22], the elements of three MMs were used to define feature vectors for the classification process. Various vector types were defined. They consisted of differently combined MM segments, belonging either to one or several selected levels, as well as included values either of one or all the analysed measures (Figure 3). However, before the creation of the vector, data from all MMs levels were rescaled using min-max normalization:

(16) $$x_{new}=\frac{x-x_{min}}{x_{max}-x_{min}}$$

Finally, the following sets were prepared (see Figure 3 for examples):

1. features based on one level (X={64, 128, 256, 512})
  - setX_ApEn, setX_FuzEn, setX_LLE, including one feature
  - setX_ApEn_FuzEn_LLE, including three features
2. features based on two levels (X_Y={64_128, 128_256, 256_512})
  - setX_Y_ApEn, setX_Y_FuzEn, setX_Y_LLE, including two features
  - setX_Y_ApEn_FuzEn_LLE, including six features
3. features based on three levels (X_Y_Z={64_128_256, 128_256_512})
  - setX_Y_Z_ApEn, setX_Y_Z_FuzEn, setX_Y_Z_LLE, including three features
  - setX_Y_Z_ApEn_FuzEn_LLE, including nine features

The number of classes ($N_{cl}$)—the number of segments at the lowest level in the given feature vector —differed in particular feature vectors, depending on the segment used ([22]):

(17) $$N_{cl}\left(setX\_Y\_Z\right)=\frac{N_{EMr}}{N_{ss}\times 2^{l_{X}-1}}$$

where $l_{X}$ is the map level and X=min(X,Y,Z). For example, if the set64_128_256 is considered: X=64 and $l_{X}=1$. This means that for $l_{X}$ equal to:

- 1 – 16 classes were defined,
- 2 – 8,
- 3 – 4,
- 4 – 2.

These sets were separately used for feeding the *kNN* classifier, run with several values for the *k* parameter: k={3,7,15,31,63,127,255}. Each feature vector set was divided into training and test sets, with the usage of the *leave-one-out cross-validation* approach: in each classifier run, $N_{pp}$ maps—calculated for one participant and for one session—were always left for defining a test set.

## 3. Results

**Figure 4:**
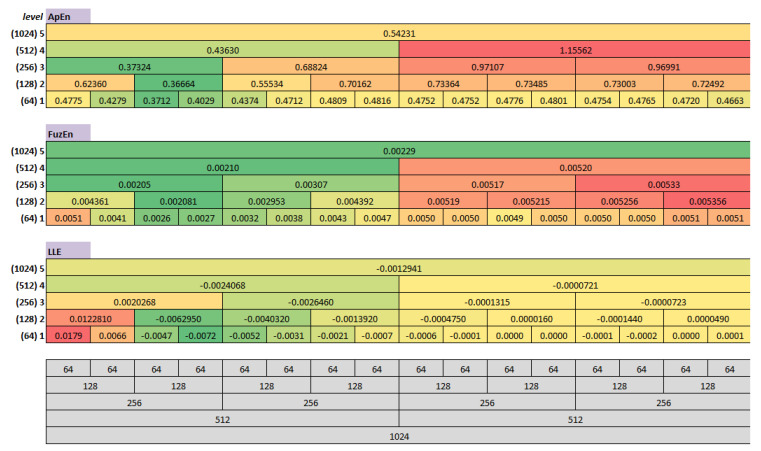
The multilevel maps obtained for the three measures: ApEN, FuzEn, the LLE, with values averaged for all EM series. The table at the bottom presents the maps’ structure.

The multilevel maps were estimated for each EM series and each analysed measure separately. Subsequently, they were averaged over all $N_{EMs}$ series, and finally, three resulting maps were created, as shown in Figure 4.

**Figure 5:**
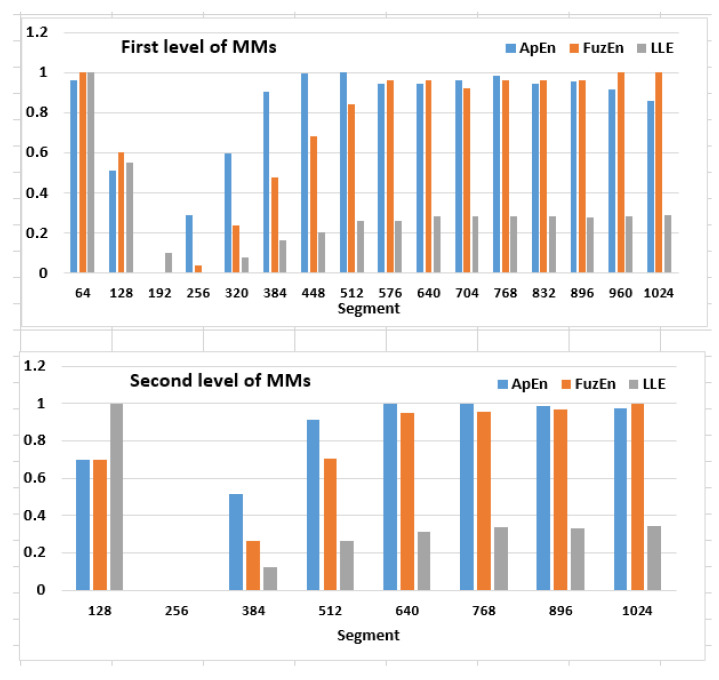
The charts presenting values from the two levels of the ApEn, FuzEn and LLE maps.The first level at the top and the second at the bottom.

The maps cells were coloured according to their contents. Green indicates the lowest values, while red the highest ones. A repeating pattern can be noticed in each MM, in the 3rd and the 4th segments at the first level and the 2nd segment at the second one. These cells contain the lowest values calculated for each measure and relate to 128 ms of eye movement signal commencing with 128 ms. This dependency is easily noticeable in Figure 5, where the results obtained for the first two MMs levels are juxtaposed in the charts. However, to make this comparison feasible, the results had to be rescaled with the min-max normalization (Equation (16)).

Statistical significance of the outcomes was checked by means of ANOVA and Tukey’s Honest Significance Difference tests. The two result sets—for ApEn and the LLE measures—verified by the Shapiro–Wilk test, turned out to be normally distributed, yet for the third one—FuzEn—the null hypothesis was rejected. In this case, the Kruskal-Wallis test was additionally run. All differences in the results obtained for the 3rd and the 4th times scopes at the first MM level, compared to the remaining set of segments turned out to be significant. However, there was one exception for the LLE—the difference between the 3rd and 5th segments—and a few for FuzEn—the differences between the 3rd, 4th, and 5th time scopes—which occurred not to be significant. While analysing the results for the second level of the maps, it was revealed that all measures provided significant differences for the 2nd segment when collated with all other time scopes. On the contrary, the outcomes from the second part of the EM series—starting from the 6th (ApEn) and the 8th (FuzEn and the LLE) segments at the first MM level, as well as from the 5th segment at the second level—disclosed the lack of significant differences.

**Figure 6:**
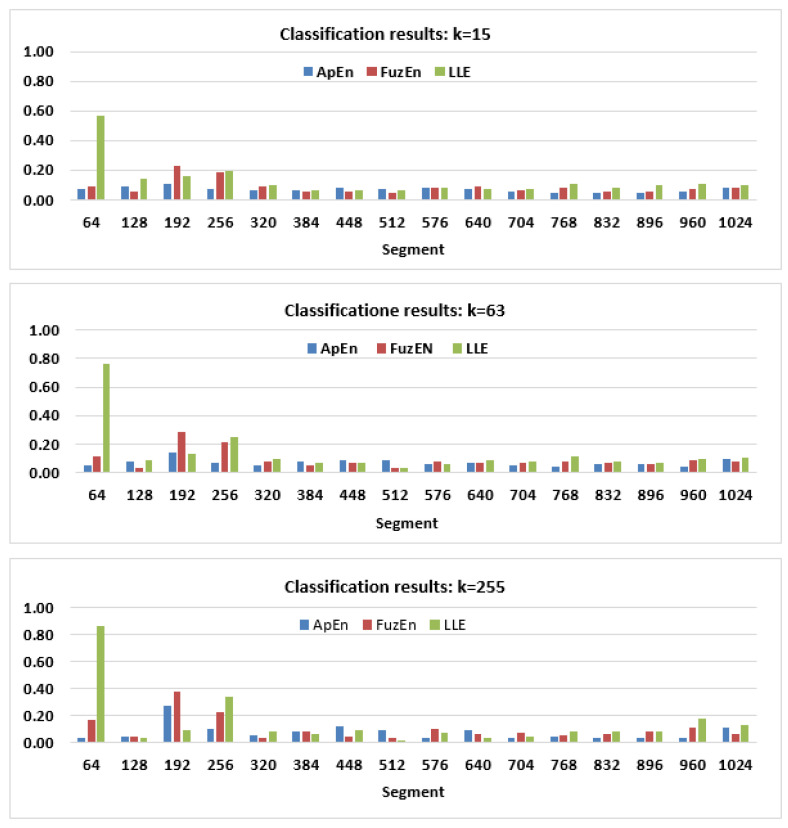
The classification accuracy based on the first level of the ApEn, FuzEn and LLE multilevel maps, and for different the k–parameter values: k = 15 (top), k = 63 (middle), k = 255 (bottom).

In the second stage of the tests, the usefulness of the three MMs in recognizing eye movement segments was verified with the usage of the kNN method. During the first classification runs, feature vectors consisting of one, two, or three elements calculated only for one measure, were used. Because the attention was focused on the highest possible signal resolution, only features based on segments 64 and 128, and their combinations with values from other levels were taken into account. As can be seen in Figure 6, the performance of the segment recognition is rather low, independently of k−value used, especially when time scopes later than 256 ms are considered. Thus, in further analysis, only the first four segments were addressed.

The exploration of the presented charts revealed that a higher *k* parameter did not always provide better results. For this reason, the attention was focused on k−value equal to 63, for which the classification accuracy was similar to that, disclosed for k=255, and which ensures better performance of the execution.

The effect of the classification with the usage of feature vectors defined based on one measure, yet using several levels of the MMs is presented in Table 1. There are nine sets taken into account: three consisting of one feature: *set64_[ApEN, FuzEN, LLE]*, three with two elements: *set64_128_[ApEN, FuzEN, LLE]*, and three including three components: *set64_128_256_[ApEN, FuzEN, LLE].*

The table content shows that the best results were obtained for the LLE measure in the first segment of eye movement signal, yet less than that got for k=255 (86% see Figure 6). However, its classification accuracy decreases significantly when other time scopes are considered. The opposite situation takes place for the two remaining groups of sets. As can be observed, both entropy measures at the beginning of the EM series revealed a low accuracy for vectors defined with only one element. The performance increases for later eye movement scopes when features are merged into two- or three-elements vectors (*set64_128_*, *set64_128_256_*).

The individual measure outcomes can be further collated with those presented in Table 2, obtained for the mixed feature vectors.

Similarly, the best performance, slightly worse than previously, was achieved for the first 64 ms of the signals, classified with samples coming from the first level of the MMs. Subsequently, the accuracy decreases, especially for the *set64_ApEN_FuzEn_LLE*. Thus, *set64_128_256_ApEN_FuzEn_LLE* seems to be the proper choice for the classification purpose, despite lower than the best accuracy produced for the first segment. It provides the best or close to the best efficiency for all analysed segments.

In the next two Table 3 and Table 4, outcomes for the subsequent segment size (128 ms) were shown. They, in terms of the result pattern, are similar to those discussed above. The best detection was delivered by the LLE measure in the first 128 ms. In the further time scope, the efficiency of this measure dropped, and ApEn and FuzEn disclosed better performance; the best for vectors with three features *(set64_128_256_ApEn*, *set64_128_256_FuzEn*). While comparing the classification accuracy to the previous results (for 64ms segment), it turned out to be generally higher. However, it should not be surprising, because of the less by half the number of classes in a feature set, which facilitates the sample recognition. Additionally, once again, the *set128_256_512_ApEn_FuzEn_LLE* despite worse efficiency in the first time scope, provides, on average, the best results.

The last step in the data exploration was the juxtaposition (Table 5) of the best results obtained in [22], using only ApEn (the third column), with the corresponding to them, achieved in these studies with the usage of three measures (the fourth column). The first column in the table represents the feature vector type, while in the second one, the eye movement segments were included. The last column in this table shows the best classification accuracy received in these studies for a feature set displayed in the first column and for the segment from the second one. All values presented in this column were estimated for the LLE measure in the first EM series scope.

In almost all cases, *Current accuracy* exposes better or similar performance; however, in one case (set64_128), the worse accuracy was achieved.

## 4. Discussion

**Figure 7:**
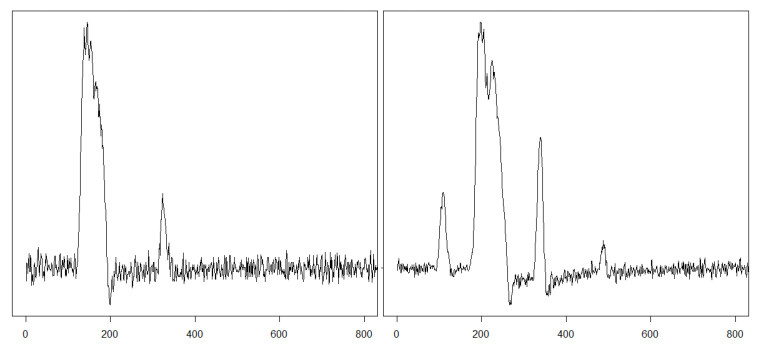
Example signal courses for eye movement velocity. For two more, refer to [22].

Eye movement analysis has been conducted over many years, but research in this domain intensified over the last ten years. It resulted from the fact that acquiring knowledge concerning visual patterns has proved useful in many areas, because they provide essential data regarding people’s behaviour, intentions, and interests. Proper reasoning in these fields requires an appropriate interpretation of collected recordings, which corresponds to correctly recognised eye movement events. Elements that constitute visual patterns are fixations and saccades, and extracting them adequately from the eye movement signal is crucial. For many years, approaches based on spatial recordings dispersion and velocity have been used. However, as mentioned, they rely on user-defined thresholds, which is their disadvantage as most of the biological patterns vary between individuals (Figure 7). Therefore, some steps were taken to search for other algorithms, ensuring more adjusted event detection.

The studies presented here base their research on the dynamical aspects of eye movement signal. The main purpose of these investigations was to find characteristics of the particular signal parts, short enough to enable searching for the smallest eye movement events, saccades.

### 4.1. Multilevel Maps Analysis

The results presented in Figure 4 showed that measures calculated for one EM series, including three eye movement events—saccadic latency, saccade and fixation—have different values in various time scopes of the EM series. The previous studies [18,19,21,22] already provided some evidence in this regard for ApEn, SampEn, and the LLE; however, only during independent analyses. One of the advantages of the current studies is a comparison of these measures and introducing the additional signal description through the FuzEn usage.

**Figure 8:**
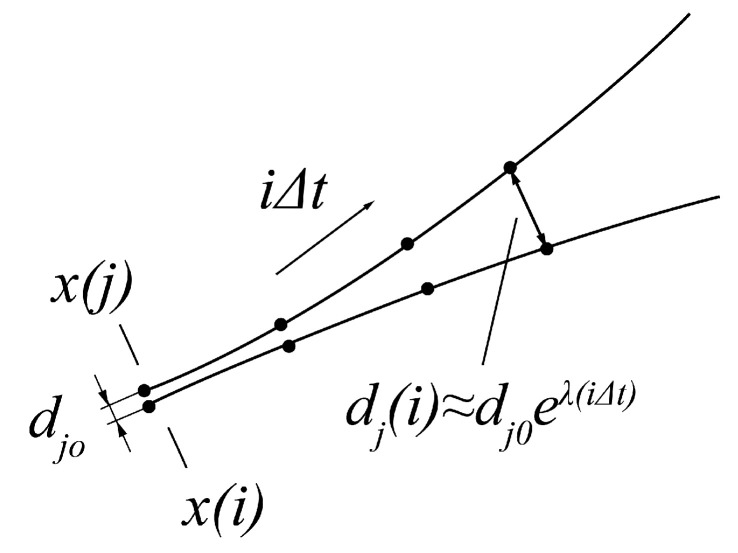
Example of divergent paths.

When analysing the three multilevel maps from Figure 4, it may be noticed that two segments at the first level (128–192 ms and 192–256 ms) and one segment at the second level (128–256 ms) include the lowest values among all those calculated for the particular map. This means that all three measures detected, within the same period, an activity different than in the remaining EM series parts. This period is characterized by lower entropy values and a lack of chaotic behaviour. Collating MMs segments with signal recordings, for example, in Figure 7 shows that this period corresponds to the saccade. Further exploration of this figure unveils another distinction visible in the LLE map, in its first two segments at the first two levels. The positive values in this period show that neighboring points of EM series, which start close to one another, diverge with time approximately at a rate given by the LLE, as shown in Figure 8.

Such behaviour characterises chaotic dynamics, which, as it was revealed in [18,19], is related to saccadic latency. Fixations provide more homogeneous results than the saccadic latency and saccade, which is visible in the second part of the time series (512–1024 ms). The segments representing this component contain comparable values in the case of each of the studied metrics.

The above analysis demonstrate that given the presented MMs, it is feasible to indicate time series scopes within which three eye movement events take place.

### 4.2. Classification Performance

Promising visual inspection of the MMs found also a partial confirmation in the classification results, although overall accuracy was rather low. This outcome is a consequence of the fact that at least half of the samples—corresponding to a fixation—have very similar characteristics. It is especially visible in the charts presented in Figure 6. However, this is not the only reason, as for saccadic latency and fixation the similar entropy values were estimated (see Figure 4). Therefore, entropy metrics, at the lowest level of the MMs, do not introduce the distinction among these two events. The situation changes at the second level, where the fixation parts differ more noticeably from the first signal scopes.

Analysis of the results presented in Table 1 and Table 3 shows that if saccadic latency is being searched for, the best approach is the usage of the LLE measure, for which accuracy at levels 76% and 84%, respectively, were achieved. Taking this event duration into account (approximately 120-200 ms), the segment of size equal to 128 ms appears to be sufficient. The advantage of the LLE over ApEn and FuzEn results from the fact that, while all three metrics quantifies irregularity in a time series, the first one is also able to describe how a system evolves in a particular period of its evolution. For saccadic latency segments, it indicates divergence, whereas during saccade, convergence. In the case of fixation, behaviour changes from convergent to chaotic and conversely. Therefore, this quantity is less efficient in fixation scopes, in which both entropy types yielded interchangeably better results. Yet, changing the measure during the classification process, depending on the segment under consideration seems not to be a proper solution. Thus, it is more reasonable to apply a feature vector consisting of all measures. Confirmation for such an approach provides the analysis of the third columns in Table 2 and Table 4. The accuracy achieved for the combination of ApEn, FuzEn and the LLE was better than for any quantity except for the LLE. The decrease in its efficiency was approximately 3% for the longer segment (128 ms) and 10% for the shorter one (64 ms).

Generally, more satisfying classification outcomes were obtained for feature vectors with samples based on 128 ms-segments (Table 3 and Table 4). As was previously noted, one of the reasons is the lower number of classes in the training and testing sets. However, it may also stem from the fact that for longer segments, entropy values for different eye movement events are more distinguishable.

Finally, the comparison between findings presented in [22] and provided here indicates that the usage of more than one measure may advance the events detection efficiency (Table 5). It especially refers to the shorter segments at the beginning of the eye movement signal. Thus, by applying the presented approach, better classification has become possible for the higher resolution of signal segments, even when using fewer than ten features. The high efficacy of the saccadic latency detection facilitates recognizing the following saccade, while finding saccade period opens the possibilities for more detailed exploration in this scope.

However, the results of this method still need further improvement, which can be achieved by, for example, extending the feature vector and usage of other classifiers. Additionally, decreasing the number of fixation segments is worth considering. In this research, the studied fixation period was approximately 700 ms and could be shortened by 200–300 ms. Furthermore, within the explored eye movement data, there was a lack of movement called *smooth pursuit*, when the eyes follow a moving object. The dynamics of such a motion should also be explored.

The suggestions mentioned above are plans for future work. Moreover, because the proposed method was verified only for one dataset gathered with the usage of a particular eye tracker type, other signal recordings are intended to be analysed.

## 5. Conclusions

The methods for nonlinear time series analysis were used in the presented research in order to reveal eye movement signal characteristics. The primary motivation for these investigations was to find patterns, which can characterise the main components of eye movements: fixation, saccade and saccadic latency. Their proper recognition in registered eye movement signals supports studies in many areas using eye tracking technology. Fixations uncover the gazed areas of observed scenes, while saccades reveal changes in gaze positions. Sometimes, an eye movement is caused by the motion of an object belonging to the scene. Such a situation introduces an additional period in the signal called saccadic latency: the time needed for the brain to react to the motion of the observed stimulus. Discovering this period is the indication that the subsequent signal segment will represent a saccade in the direction of a new stimulus position. Recognizing this element is, in turn, equivalent to revealing the subsequent fixation. Although there are some commonly used methods for eye movement events detection, new solutions, which will provide more accurate event detection, are still being sought.

In the presented approach, three measures were used: approximate entropy, fuzzy entropy and the Largest Lyapunov Exponent, for which multilevel maps, being their time-scale decomposition, were defined. To check if the estimated characteristics may be useful in distinguishing eye movement components, these structures were applied in the classification process. It produced an improvement in the accuracy, for saccadic latency and saccade, when compared to previously conducted studies using eye movement dynamics. Nonetheless, further investigations towards more precise results are planned in the future.

## Figures and Tables

**Table 1:** The percentage of the correctly classified samples for various feature vectors defined with the usage of one measure. The smallest segment size = 64 ms.

Vectors		set64_			set64_128_			set64_128_256_
**Segment**	**ApEn**	**FuzEn**	**LLE**	**ApEn**	**FuzEn**	**LLE**	**ApEn**	**FuzEn**	**LLE**
1–64	0.05	0.12	0.76	0.07	0.13	0.63	0.25	0.34	0.71
64–128	0.08	0.04	0.09	0.07	0.05	0.53	0.34	0.29	0.60
128–192	0.14	0.29	0.13	0.39	0.29	0.26	0.38	0.30	0.40
192–256	0.07	0.21	0.25	0.42	0.25	0.27	0.42	0.28	0.40

**Table 2:** The percentage of the correctly classified samples for the feature vectors defined with the usage of three measures: ApEN_FuzEn_LLE. The smallest segment size = 64 ms.

Segment	set64_	set64_128_	set64_128_256_
1–64	0.74	0.61	0.66
64–128	0.27	0.38	0.57
128–192	0.10	0.52	0.60
192–256	0.23	0.38	0.51

**Table 3:** The results–the percentage of the correctly classified samples for various feature vectors defined with the usage of one measure. The smallest segment size = 128 ms.

Vectors		set128_			set128_256_			set128_256_512_
**Segment**	**ApEn**	**FuzEn**	**LLE**	**ApEn**	**FuzEn**	**LLE**	**ApEn**	**FuzEn**	**LLE**
1–128	0.08	0.05	0.84	0.52	0.48	0.80	0.60	0.49	0.82
128–256	0.72	0.64	0.48	0.67	0.61	0.57	0.80	0.71	0.59
256–384	0.07	0.16	0.23	0.30	0.32	0.45	0.60	0.42	0.49
384–512	0.17	0.08	0.14	0.32	0.41	0.47	0.42	0.45	0.54

**Table 4:** The results–the percentage of the correctly classified samples for the feature vectors defined with the usage of three measures: ApEn_FuzEn_LLE. The smallest segment size = 128 ms.

Segment	Set128_	Set128_256_	Set128_256_512_
1–128	0.78	0.81	0.81
128–256	0.69	0.75	0.79
256–384	0.20	0.52	0.61
384–512	0.20	0.53	0.66

**Table 5:** The best accuracy obtained in [22] and in the current research for the same feature vector types and the same EM series segment.

Set	Segment	Accuracy	Current	Current
		[22]	accuracy	the best
		(ApEn)	(ApEn_FuzEn_LLE)	(LLE)
set64	128–192	0.19	0.27	0.76
set64_128	192–256	0.46	0.38	0.63
set64_128_256	128–192	0.43	0.57	0.71
set128	128–256	0.72	0.69	0.84
set128_256	128–256	0.71	0.75	0.80
set128_256_512	128–256	0.83	0.80	0.82

## Abbreviations

The following abbreviations are used in this manuscript:

**Abbreviation**	**Description**	**Value**
$N_{pp}$	Number of point positions	29
$N_{p}$	Number of participants	24
$N_{spp}$	Number of sessions per participant	2
$N_{ps}$	Number of participant sessions	44
$N_{EMs}$	Number of eye movement series	1276
$N_{EMr}$	Number of eye movement recordings	1024
$N_{ss}$	Number of samples in the smallest segment of eye movement recordings	64
